# The Utility of Augmented Reality in Spinal Decompression Surgery Using CT/MRI Fusion Image

**DOI:** 10.7759/cureus.18187

**Published:** 2021-09-22

**Authors:** Ryoma Aoyama, Ukei Anazawa, Hiraku Hotta, Itsuo Watanabe, Yuichiro Takahashi, Shogo Matsumoto

**Affiliations:** 1 Orthopaedics, Tokyo Dental College Ichikawa General Hospital, Chiba, JPN

**Keywords:** mixed reality, foraminotomy, head-mounted display, hololens, spine, fusion image, augmented reality, holoeyes

## Abstract

In spine surgery, instrumentation surgery using augmented reality (AR) and navigation systems have become widespread, while decompression surgery using those applications is not so common. However, we sometimes encounter intraoperative problems such as excessive blood loss or bony resection in decompression surgery. Therefore, a practical navigation system is needed for safer spinal decompression surgery. Furthermore, the cost of AR and navigation systems has been expensive. In this study, we report the utility of applying the AR system of the head-mounted display (HMD) at a lower cost to identify the osteotomy area of laminectomy for spinal decompression surgery.

3D CT/MRI fusion images are created preoperatively to generate 3D data consisting of the nerve elements, a dural tube and nerve roots, and the bony elements of the spine. Then, we made the 3D data of the bone after decompression by 3D editing free software. Uploading the created 3D data of both 3D CT/MRI fusion and preoperative planned laminectomy images to the AR software in the HMD, we could confirm the proper decompression area with the 3D images projected through the HMD. This system was useful for cervical and lumbar decompression for confirming the proper decompression area preoperatively.

We could perform decompression surgery just designed with this system. This system is a preoperative planning system that allows 3D HMD visualization to keep track of surgical orientation. It does not allow preoperative verification so far. However, this system has various possible applications and is considered a promising system for the future.

## Introduction

As personal computers and augmented reality (AR) equipment performance improve, it is becoming easier to handle 3D images at home. In the medical field as well, various AR devices have been used recently [1, 2].

Up to now, plain radiograph, CT (computed tomography), MRI (magnetic resonance imaging), etc. are evaluated on a 2D display, even if they are reconstructed into 3D images. When evaluated, a 3D image must be assessed on the monitor that can only display in 2D, so the data is likely reconstructed in 3D in the brain of the evaluators. They are forced to use their imagination to evaluate the images, especially for missing parts not captured in the 2D slices, such as severely compressed nerve root area on MRI.

The AR device eliminating the need for this reconstruction in the brain can enable us to evaluate 3D data as it is in 3D. By displaying the 3D data in true 3D using a head-mounted display (HMD), the 3D data can be intuitively grasped in the brain. This 3D display makes it possible for new learners to evaluate imaging data quickly. In addition, patients will be able to understand their condition quickly and accurately.

Recently, creating fusion images of CT and MRI has become a standard feature of many workstations in hospitals, and it is easier than ever to create such fusion images [3, 4]. It is now possible to create more sophisticated 3D images with fusion images composed of the data of CT for bony images and MRI for the spinal cord and nerve roots [4]. In addition, even a personal computer can edit the obtained 3D data recently. The environment for using 3D data in the medical field is becoming increasingly ready.

The navigation systems of spinal decompression surgery have not been well established compared to those of spinal instrumentation surgery [1, 2]. However, we sometimes encounter intraoperative problems such as excessive blood loss or bony resection. Therefore, a practical navigation system is needed for safer spinal decompression surgery.

In this study, we would like to illustrate the 3D image displayed on the HMD of preoperative spinal structures including both bone and nerve elements and of decompressed spine created from preoperative surgical planning using Holoeyes MD, a software certified as a medical device in Japan, and describe its usefulness of identifying proper decompression area.

## Technical report

Applied cases

Case 1

**Figure 1:**
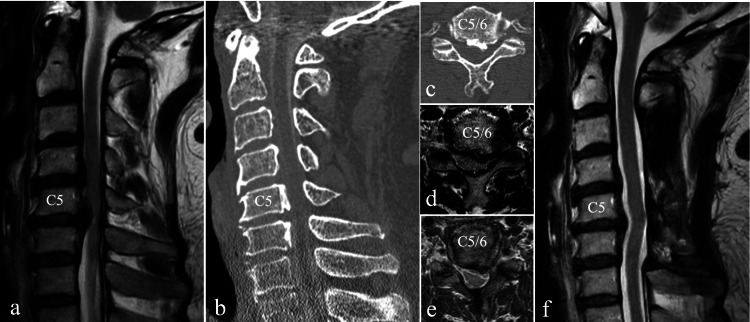
Preoperative MRI (a, d), CT (b, c), and postoperative MRI (e, f) of case 1 a. Preoperative MRI T2-weighted sagittal section showed the spinal canal stenosis from C3 to C6, with severe spinal cord compression at C5/6. b. Preoperative CT myelography sagittal section showed the ossification of the posterior longitudinal ligament at C4 to C6. c. Preoperative CT myelography transverse section of C5/6 showed compression of the left C6 nerve root due to the ossification of the posterior longitudinal ligament. d. Preoperative MRI T2-weighted transverse section of C5/6 showed compression of the left C6 nerve root and spinal canal stenosis. e. Postoperative MRI T2-weighted transverse image showed that the left C6 root and spinal canal were decompressed by laminectomy and foraminotomy. f. Postoperative MRI T2-weighted sagittal section showed that the spinal cord was decompressed.

A 62-year-old man came to our hospital with left upper extremity pain and clumsiness of the hand that had been refractory to conservative therapy for two months. MRI revealed severe stenosis at C5/6 and narrowing of the spinal canal at the C3 to C6. CT myelography showed ossification of the posterior longitudinal ligament at the C3 to C6. The patient underwent a laminectomy of C3 to C6 and a foraminotomy of the left C5/6. A four-month postoperative MRI showed an enlargement of the spinal canal and intervertebral foramen. The pain in the left upper extremity was markedly improved, and the patient's clumsiness of the hand was reduced (Figure 1).

Case 2

**Figure 2:**
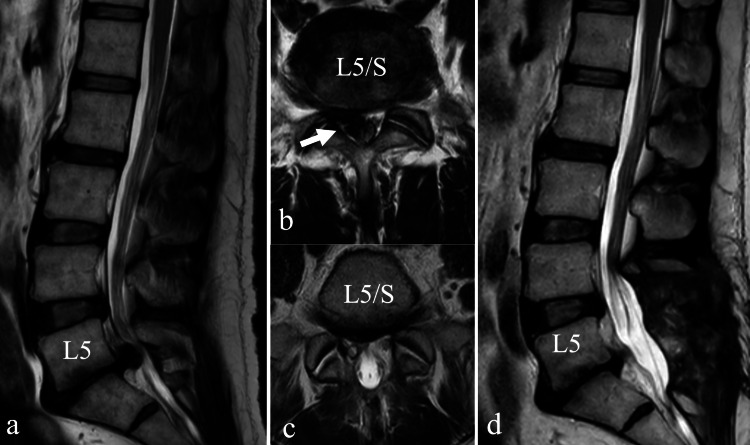
Preoperative (a, b) and postoperative (c, d) MRI of case 2 a. Preoperative MRI T2-weighted sagittal section showed stenosis at L4/5 and a large herniation protruding at L5/S. b. Preoperative MRI T2-weighted transverse image showed a shift of the dural tube to the left due to the herniation (arrow) that prolapsed to the right at L5/S. c. Postoperative MRI T2-weighted transverse section showed the disappearance of the herniation, widening of the dural tube, and the decompressed right S1 nerve root. d. Postoperative MRI T2-weighted sagittal section showed that the stenosis of L4/5 and 5/S had been decompressed, and the hernia had disappeared.

A 54-year-old man with a history of lower extremity pain for several years came to our hospital with an exacerbation of right lower extremity pain after a fall three months earlier, with no improvement despite medication and nerve root block. He came to our hospital because his right lower extremity pain was marked, and he had to use a cane to walk. MRI revealed a massive herniation with significant protrusion to the right at the L5/S1 disc and stenosis at L4/5. The patient underwent decompression with L4 partial laminectomy and L5 laminectomy, and L5/S disc herniotomy. Postoperative pain in the lower extremities markedly improved, and pain on walking disappeared. A two-month postoperative MRI showed that the stenosis had been decompressed, and the herniation had disappeared (Figure 2).

Technical note

CT images were taken using Aquilion one vision edition (Canon Medical Systems Corporation, Tochigi, Japan) or Brilliance 64 CT scanner (Philips Japan, Tokyo, Japan).

MRI images were taken using an Ingenia 3.0 T (Philips Japan, Tokyo, Japan). A 3D STIR sequence was used to generate 3D data of the dural tube and nerve roots in the cervical spine [5], and a T2 FFE PROSET sequence was used in the lumbar spine [6]. CT/MRI fusion images were created using a workstation (Zio station 2 plus; Ziosoft Corporation, Japan).

**Figure 3:**
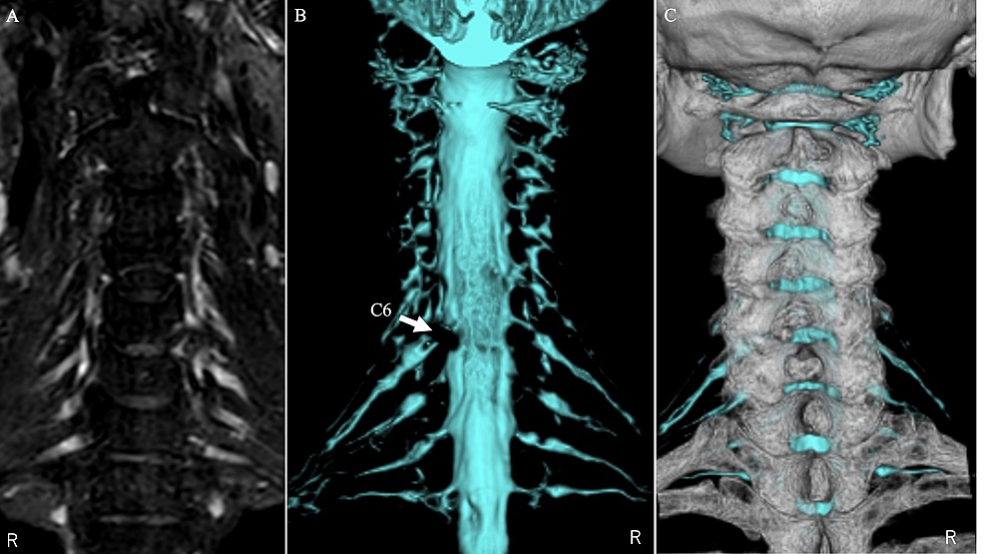
CT/MRI fusion images of case 1 The left image (A) shows the nerve root in the 3D STIR sequence. The middle image (B) is a 3D MRI image of the dural tube and the nerve root taken with a 3D STIR sequence on the left image. The right image (C) is a CT/MRI fusion image of the 3D CT image and the 3D MRI image.

**Figure 4:**
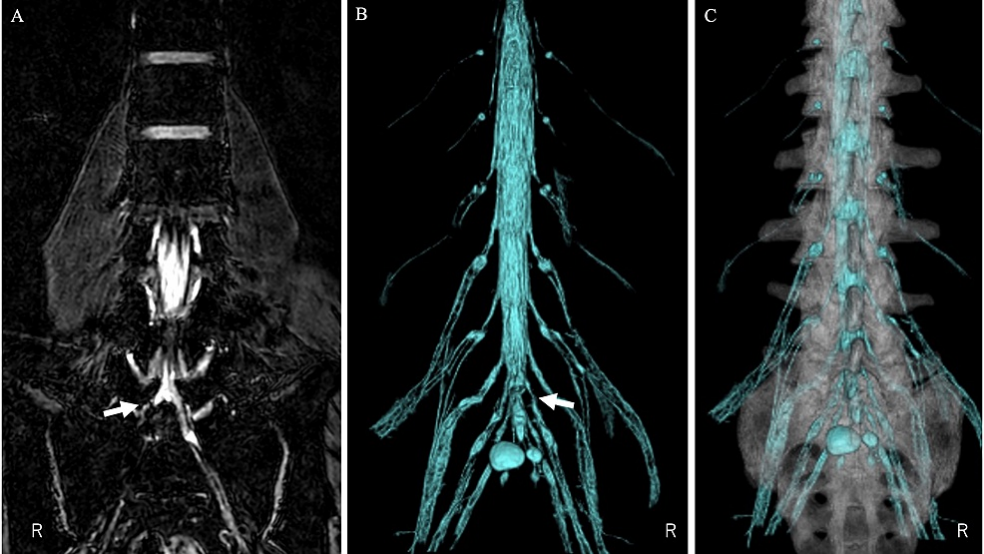
CT/MRI fusion image of case 2 The left image (A) shows the nerve root in the T2 FFE PROSET sequence. The S1 nerve root is disrupted by the herniation (arrow). The middle image (B) is a 3D constructed MRI image of the dural tube and the nerve roots in the T2 FFE PROSET sequence on the left image. The dural tube and nerve root are partially lost due to herniation at the arrow. The right image (C) is a CT/MRI fusion image of a 3D CT image and a 3D MRI image.

When differences in location or angle appeared between the CT and MRI data, the CT and MRI images were checked individually and fine-tuned to achieve the best consistency near the lesion site. The STL data of bony elements and STL data of the dural canal and nerve roots were then output by a workstation (Figures 3, 4).

**Figure 5:**
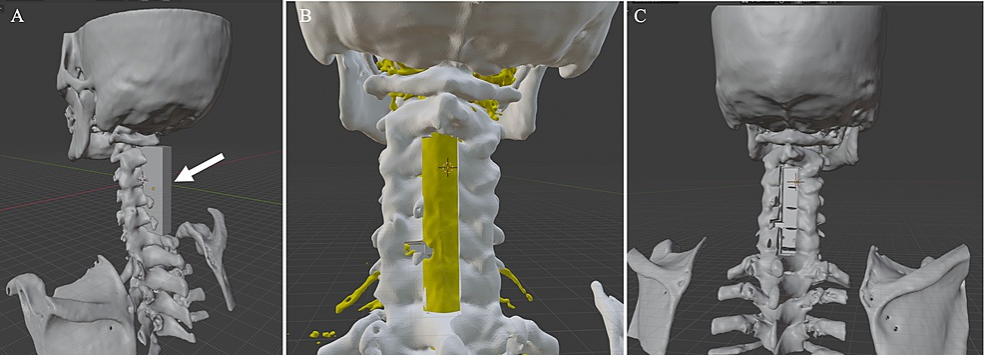
The creation of a laminectomy model using the 3D image editing software Blender On the left picture (A), the cuboid (arrow) is used to create the laminectomy model. The spinous processes and the laminae that overlap the cuboid are removed. On the center picture (B), the lateral edge of the nerve root defect shadow can be used as a reference to determine the lateral edge of the intervertebral foraminotomy. The bone removal is performed by fabricating a cuboid of any size (any number of small units can be specified) in the same manner as in picture A. On the right picture (C), a model with C3-C6 laminectomy and left C5/6 foraminotomy is created. This model is output as STL data.

3D image editing free software (Blender; Blender Foundation, Amsterdam, Netherlands) was used for the STL data of bony elements. The preoperatively planned decompression area is deleted from the original bone data using 3D image editing software to create STL data of the expected laminectomy image for decompression surgery. The outer edge of the intervertebral foraminotomy could be determined by referring to the lateral edge of the nerve root shading defect (Figure 5).

**Figure 6:**
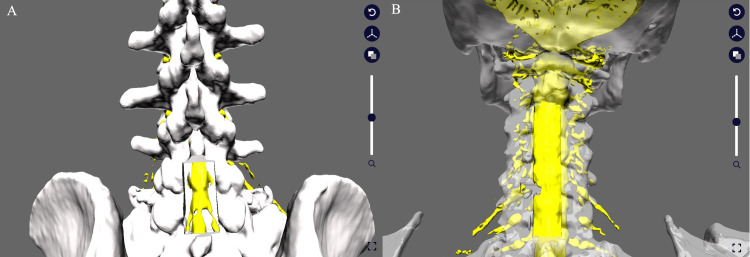
Uploading STL data to the Holoeyes MD system The STL data are uploaded using a web browser. On the left (A) are the data from Case 2 upload, and on the right (B) are the data from Case 1 upload. On the right (B), the transparency of the bone model after decompression has been increased to make it easier to identify the nerve. The uploaded STL data will be reconstructed for Holoeyes MD in a few minutes, which can then be downloaded via the Internet using the Holoeyes MD software in the head-mounted display to view the created polygonal images.

**Figure 7:**
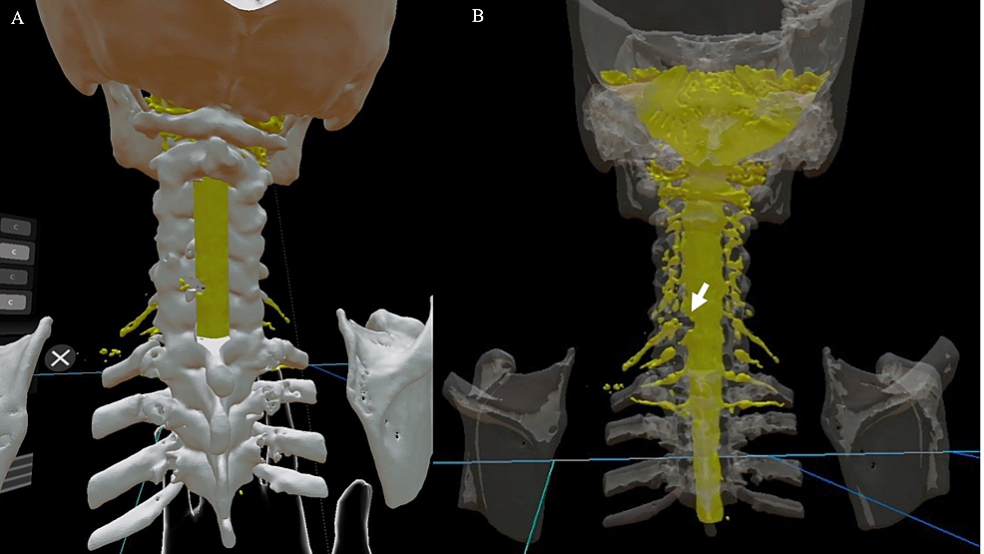
Case 1 in the Holoeyes MD software On the left (A) is the posterior view after C3-C6 laminectomy and the left C5/6 foraminotomy. In the right image (B), the bone transparency is increased to make it easier to identify the nerve, which makes it easier to confirm the positional relationship between the nerve compression sites (defect indicated by the arrow) and the surrounding bone and confirm whether the decompression site is appropriate in this model. The actual 3D image is more intuitive and easier to grasp because it can be seen in 3D.

**Figure 8:**
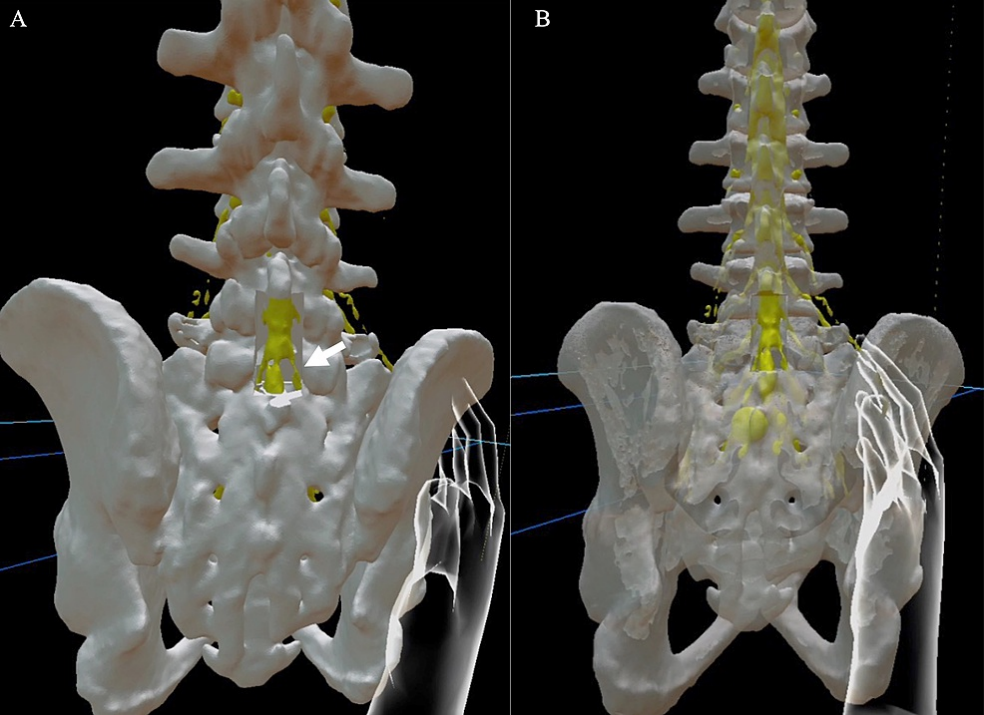
Case 2 in the Holoeyes MD software On the left (A) is the posterior view after L5 laminectomy and L4 and S1 partial laminectomy. The width of the laminectomy was set at 19 mm. The arrow indicates the position of the safe approach to the herniation. On the right (B), the bone is more transparent so that the location of the nerve can be easily identified. The actual 3D image is more intuitive and easier to grasp because it can be seen in 3D.

These three data, the original bone, nerve elements data, and the expected laminectomy image data are displayed on the HMD, an augmented reality device (HoloLens 2; Microsoft Corporation, Redmond, Washington) using a dedicated commercially available application (Holoeyes MD; Holoeyes Corporation, Japan). Holoeyes MD can arbitrarily hide each uploaded 3D image, bony or nerve elements, or adjust the transparency of each image for evaluators (Figures 6-8).

Cases in the HMD system

Case 1 (Cervical Case)

A 3D model of the dural tube and nerve roots revealed the left C6 nerve root defect that is shown only as missing parts in the 2D slices. The left C6 nerve root position can be estimated from the shade of the lateral side of the non-defective nerve root and be identified by the location of the other nerve roots. Fusion images of nerve and bone elements demonstrated accurate compressive nerve root position in the bone elements (Figure 3).

A 3D osteotomy model on the missing C6 nerve root was created by comparing the position of the nerve root to bone elements in the 3D model, which enables us to evaluate the 3D image of the pathological condition including the relationship between nerve and bone elements. Identifying the site of nerve root compression from this 3D model clarified the accurate bone decompression area (Figure 7). This precise bone decompression could prevent uncontrolled bleeding of blood vessels adjacent to the nerve root.

Case 2 (Lumbar Case)

On the fusion 3D image, the location of the disc herniation was identified, and the proper amount of osteotomy to the lateral side of L5 could be decided (Figure 4).

Since the positional relationship between the right S1 nerve root and the herniation could be clarified, we could plan how to remove the herniation from, such as the nerve root shoulder or axilla, which allows us to perform an atraumatic maneuver on the nerve root. In this example, the position of the missing right S1 nerve root could be inferred from the position of the left S1 nerve root (Figure 8).

## Discussion

This system can be regarded as a navigation system in the larger sense of spine surgery. The development of navigation systems in the field of spinal instrumentation has been remarkable [1, 2], and it is assumed that the fierce competition among instrumentation manufacturers has contributed to the development of this field. However, the application of navigation systems in spinal decompression surgery is rare [1, 2].

Careful preoperative planning will contribute to a reduction in the amount of soft tissue resection and blood loss. Identifying the location of the nerve root could contribute to the atraumatic manipulation of the nerve. When removing a large disc herniation, we can identify the location of the herniation and the nerve root with this system to estimate the appropriate site for removing the herniation.

In the case of cervical foraminotomy, bleeding from the blood vessels accompanying the nerve roots could often occur. It may take time to stop the bleeding. This condition would make it difficult to confirm the decompression sufficiently [7]. Preoperative accurate evaluation of bone removal area and nerve root position can allow bone removal with less touching the blood vessels, which could reduce bleeding. In addition, 3D CT/MRI fusion images could be used to help determine the lateral edge of the foraminotomy. This needs to be verified in the future with more cases. In the case of lumbar herniotomy, the advantage of planning the decompression site is reducing unnecessary bone resections and avoiding nerve damage by planning how to handle the dural tube and nerve roots.

This system is useful for preoperative planning of spinal surgery, but it may also be useful for diagnosing conditions that are easily misdiagnosed, such as external lumbar spinal stenosis. An article points out the usefulness of CT/MRI fusion images for lateral lumbar spinal stenosis, which may be even more useful along with this system [3].

It has been reported that the use of AR technology as an intraoperative navigation system can reduce radiation exposure during screw insertion and contribute to the safety of surgery by providing intraoperative and preoperative information on the location of vital organs such as blood vessels [1, 2]. This HMD system also seems to have a promising future for use in preoperative surgical planning and intraoperative navigation.

A problem with this system is the time required to create the 3D image data. It took about one hour to create the data for each case in this study. Additionally, it costs money to purchase an HMD and use the Holoeyes MD software, and its costs are 4,000 USD and 30,000 USD a year. Still, the price is an order of magnitude lower than the navigation systems for instrumentation surgery. In Japan, most spine surgeries performed using this system can be billed to the national health insurance as creating intraoperative support images. Although this system can be used intraoperatively for surgeries performed using the naked eye, it is difficult to use it intraoperatively when using a microscope. Since we use a microscope for spinal decompression, it is necessary to remove the microscope and check the decompression with the naked eye when we use this HMD system directly intraoperatively. For this reason, HMDs are currently used for preoperative planning, and we believe they need to be improved for intraoperative use for confirming decompression. It is necessary to consider that the body position during surgery differs from the one during MRI and CT examinations.

## Conclusions

3D CT/MRI fusion images and commercially available AR equipment at a lower cost preoperatively providing the 3D images of pathological conditions could contribute to performing spinal decompression just designed. The advantage of this system is reducing unnecessary bone resections, avoiding nerve damage, and reducing bleeding from the blood vessels accompanying the nerve roots. This system has various possible applications and is considered a promising system for the future.
